# Type B Aortic Dissection CTA Collection with True and False Lumen Expert Annotations for the Development of AI-based Algorithms

**DOI:** 10.1038/s41597-024-03284-2

**Published:** 2024-06-06

**Authors:** Christian Mayer, Antonio Pepe, Sophie Hossain, Barbara Karner, Melanie Arnreiter, Jens Kleesiek, Johannes Schmid, Michael Janisch, Deutschmann Hannes, Michael Fuchsjäger, Daniel Zimpfer, Jan Egger, Heinrich Mächler

**Affiliations:** 1 Division of Cardiac Surgery, Department of Surgery, Medical University of Graz, Auenbruggerplatz 29, 8036 Graz, Austria; 2 Institute of Computer Graphics and Vision (ICG), Graz University of Technology, Inffeldgasse 16/II, 8010 Graz, Austria; 3 Institute for Artificial Intelligence in Medicine (IKIM), AI-guided Therapies (AIT), Essen University Hospital (AöR), Girardetstraße 2, 45131 Essen, Germany; 4 Division of General Radiology, Department of Radiology, Medical University of Graz, Auenbruggerplatz 9, 8036 Graz, Austria

**Keywords:** Experimental models of disease, Aortic diseases, Aneurysm

## Abstract

Aortic dissections (ADs) are serious conditions of the main artery of the human body, where a tear in the inner layer of the aortic wall leads to the formation of a new blood flow channel, named false lumen. ADs affecting the aorta distally to the left subclavian artery are classified as a Stanford type B aortic dissection (type B AD). This is linked to substantial morbidity and mortality, however, the course of the disease for the individual case is often unpredictable. Computed tomography angiography (CTA) is the gold standard for the diagnosis of type B AD. To advance the tools available for the analysis of CTA scans, we provide a CTA collection of 40 type B AD cases from clinical routine with corresponding expert segmentations of the true and false lumina. Segmented CTA scans might aid clinicians in decision making, especially if it is possible to fully automate the process. Therefore, the data collection is meant to be used to develop, train and test algorithms.

## Background & Summary

Aortic dissections (ADs) are serious conditions of the main artery of the human body, where a tear in the inner layer of the aortic wall leads to the formation of a new channel for blood flow, named false lumen. ADs can be classified as

- Stanford Type A AD, if the disease involves the ascending aorta and may propagate to the aortic arch and descending aorta,
- Stanford Type B AD, if the disease does not involve the ascending aorta and occurs in any part of the aorta distal to the left subclavian artery)^1,2^, and
- the rare non-A non-B AD, if the proximal extent of the dissection involves the aortic arch^3^.

The wall of the aorta consists of three layers, intima, media, and adventitia. If a disruption of the intimal layer occurs, a tear causes blood to flow between the layers, creating the false lumen^4–7^. Important risk factors are arterial hypertension, atherosclerotic disease, male gender, bicuspid aortic valve and connective tissue diseases, such as Marfan and Ehler´s Danlos syndromes^1,8^. ADs can also be caused by chest trauma that results in acute increased stress against the aortic wall^1,2^.

The most common clinical symptom of an acute type B AD is sudden severe chest pain radiating to the back^1^. A CTA scan is recommended as first line imaging and can differentiate AD from other entities of an acute aortic syndrome^9^.

In contrast to highly lethal type A ADs, which are surgically managed, type B ADs are separated in uncomplicated and complicated cases. In uncomplicated cases, the first-line treatment is to manage hypertension. Also, in cases with high-risk anatomic features (aortic diameter >40 mm, false lumen diameter >20–22 mm, entry tear >10 mm, entry tear on lesser curvature, increase in total aortic diameter of more than 5 mm between serial imaging studies, hemorrhagic pleural effusion, imaging-based evidence of malperfusion) minimally invasive thoracic endovascular aortic repair (TEVAR) may be considered^1^. High-risk clinical findings are refractory hypertension despite more than 3 different classes of medications at maximal recommended or tolerated dose, refractory pain persisting longer than 12 h and need for readmission^1^. Complicated type B ADs are defined as aortic rupture (either free or contained (hemothorax, increasing periaortic hematoma or both, mediastinal hematoma), branch artery occlusion and malperfusion (with or without clinical evidence of ischemia), extension of dissection flap either distally or proximally (i.e., retrograde dissection), progressive aortic enlargement of the true, false or both lumens), intractable pain or uncontrolled hypertension^1^. In those cases minimally invasive thoracic endovascular aortic repair (TEVAR)^1,10–12^ is preferred as a worldwide standard to lower the morbidity and mortality.

To understand the complexity of TEVAR therapy it should be emphasized that there exists a broad spectrum of different TEVAR devices, which must be tailored to the patient’s individual anatomy. The optimal length, the percentage of oversizing of aortic diameters (resulting in different wall tensions), as well as the optimal timing are crucial factors for a successful treatment^1^. Additionally, exact data of the aortic geometries of the false as well as true lumen, including their biomechanical properties as well as the characteristics of entries and re-entries are important as well as the topography of the proximal landing zone^1^. Additionally, some patients with uncomplicated type B AD benefit from conservative treatment, therefore the prediction of the progression of false lumen thrombosis^13^ and aortic remodeling^11,14^ in the long-term run is crucial. For further understanding of TEVAR procedure-related risks^1^, such as the risk for inadequate proximal or distal landing zones, unplanned left subclavian artery coverage, stroke, visceral ischemic complications, spinal injury, access issues, the development of endoleaks, stent collapse, aortic perforation, bird beaking, device migration or even device rupture should be addressed^1^. In cases with short proximal landing zones, aortic adjunctive debranching procedures^1^ with different surgical debranching options might be required. TEVAR can also be performed as a second or third stage procedure in cases with progression of aortic disease, especially in cases with growing distal aortic diameters or endoleaks^1^.

High quality contrast-enhanced CTAs^15^ are necessary before and after an aortic procedure in order to diagnose and visualize the extent of the aortic disease^16^. Exact cross-sectional measurements of the thoracic aorta are important and the use of an electrocardiogram (ECG) for ECG-gated techniques can improve the assessment as the dimension changes during systole and diastole^17^.

The evaluation of a CTA^18–20^ can be done by a medical expert team of radiologists and cardiac or vascular surgeons by evaluating the scan and annotating observations and quantitative features such as aortic diameters at certain landmark positions. This, however, only results in an approximation of the entire AD anatomy. An additional three-dimensional (3D) segmentation model would not only aid and accelerate expert evaluation and complement manual measurements in CT scans^21^ but may also offer advantages for TEVAR planning, simulation of stent-graft deployment^22^ and construction of individually tailored stents in complex anatomy.

This results in a great need to develop robust computer vision algorithms and multivariate image analysis tools in order to perform optimized patient care at the primary event and to accelerate comparison of CTA follow-ups in the long-term course. The CTA analyses are time-consuming when performed manually, i.e. on a slice-by-slice basis. Numerous applications for computer-aided diagnosis tools are currently under development for research purposes and have the potential to improve clinical practice. Making data sets with ground truth segmentations publicly available will accelerate the process of creating reliable fully-automated segmentation and imaging tools.

**Potential benefits and applications of 3D-segmentation in Type B AD:**

- Accelerated segmentation: Automated segmentation algorithms trained on segmented datasets of AD would perform segmentation tasks within seconds while manual segmentation of one CTA can take up to 4–6 hours. This is not feasible in the clinical routine, therefore the integration of automatized segmentation is necessary.
- Aid in the assessment of complex morphology of type B ADs: The dissection can affect the entire descending and abdominal aorta, arterial branches can originate partially or completely from the false lumen. The length and the morphology of the primary tear causing the dissection as well as additional entry and re-entry tears have to be diagnosed as well as the extension of the flap. Uncomplicated ADs have to be differentiated from complicated ADs implying different therapeutic strategies. Aortic true and false luminal diameters and volumes must be accurately measured and progressive enlargement of the lumina on follow-up scans must be reliably detected. Evaluation of calcifications and kinking of the aorta and the detection of anatomical variants, such as an arteria lusoria, is relevant.
- Support TEVAR planning: The appropriate length of the stent-graft device (custom-made or standard models, conical stents), the degree of oversizing of the stent, evaluations of the proximal landing zone, planned left subclavian artery coverage as well as preprocedural debranching procedures are important factors of success. For this purpose a preprocedural, precise and fully automated segmentation of the complete aorta of each CTA, starting at the level of the aortic valve and including the peripheral arteries would we optimal in order to observe pathologies in diameters, true and false lumen morphologies, entries as well as re-entries.
- Simulate stent-graft biomechanics: Virtual stent-graft deployment^22^ can be simulated using a 3D model of the aorta. Incorporating stent-graft properties such as radial and spring-back forces, biomechanical graft behavior^14^ in the individual anatomy can be studied, assessing, e.g., wall stress concentrations towards the aortic wall, stretch stress relationship^14^ or shortening of the stent after implantation.
- Follow-up assessment: Upon diagnosis, patients with ADs routinely undergo CTA after 3, 6 and 12 months, and yearly follow-up thereafter. Segmentation information can help standardize and accelerate assessment of disease progression. It is crucial to detect any progression of the disease itself, and complications of TEVAR such as endoleaks types I-V^1^.
- Prediction of disease progression: Combining the anatomical 3D information from segmented ADs with outcome data, models can be developed to predict the likelihood of disease progression or false lumen thrombosis^13^. By means of computational fluid dynamics, characterization of entry/reentry morphology, rheological modeling, motion analysis^23^ thrombus growth models as well as aortic volume calculation^24^ used to gain new insights in progression and thrombus formation in ADs.
- Modeling of AD pathophysiology: Anatomical information of ADs can be combined with clinical information such as hypertension, arteriosclerosis, bicuspid aortic valve, genetic mutations. Additionally specific research questions such as flow effects of entries/reentries, shape analysis and flow simulations to evaluate the stress and mechanical loads to the aortic wall might be tested.
- Finally automated deep-learning algorithms could be used for teaching and to construct metadata to develop new devices (customized TEVAR devices, 3D-printed stents, development of biological stents).

Here, we provide a dataset^25,26^ of fully segmented CTAs of patients with type B ADs that can be used for training of segmentation algorithms or in other applications that require the segmented 3D anatomy of type B ADs.

## Methods

This study was approved by the ethics committee of the Medical University of Graz, Austria (EK-34-161 ex 21/22). Informed consent was waived due to it being a retrospective study in which anonymization was maintained with all patient specific data being removed from the uploaded files. Additionally, for the purpose of complete anonymization, the head and facial regions were cropped from CT-files including them. Data protection laws were respected according to the Austrian Data Protection Law and the Helsinki Declaration § 32.

### Data acquisition and selection

We conducted a search for CTAs in our regional hospital network that were obtained during routine clinical practice between 2005 and 2021. We included only type B ADs and excluded all type A ADs. Further excluded were CTAs with bad image quality, markedly thrombosed false lumen, pronounced motion artifacts or CTAs where a distinction between true and false lumen was not clearly possible. Distinction between true and false lumen was unequivocal in the selected AD cases and in accordance with typical radiologic imaging features.

Another issue in CT scans of ADs are artifacts and complexity of the involved structures. Artifacts in CTA scans can be caused by various factors such as motion artifacts, presence of metal implants and inadequate scan timing after contrast injection^18,19,27^. In order to achieve proper segmentations, we ruled out CTAs with excessive artifacts due to metal implants, such as patients after TEVAR or CT scans with a lot of motion artifacts. However, motion artifacts of the intimal flap often cannot be avoided and are therefore seen in some CTA scans, but we ruled out scans in which an exact differentiation between true and false lumen was not possible for more than just a few slices. To facilitate the segmentation process with regard to complexity, we ruled out CT scans in which the true and false lumen were not contrasted well. An overview of the patient specific and anatomical data is given in Table 1.

**Table 1:** Basic information like age, sex, complicated or uncomplicated dissection, CT slice thickness and anatomical information like diameters and AD shape description like kinking, helical shape and branch involvement.

Patient	Age [years]	Sex	Complicated dissection*	Morphology	CT slice thickness [mm]	∅_AscAo_ [mm]	∅_DescAo_ [mm]	Aortic kinking	Larger FL than TL	Helical shape	Interluminal gap	≥1 abdominal branch from FL	AD into iliac arteries	AD segmentation complexity
1^∇^	50	male	no		2.0	36	29	no	yes	no	no	yes	yes	simple
2^∇^	61	female	yes		2.0	34	34	no	yes	no	no	yes	yes	simple
3^∇^	53	male	yes		2.0	37	45	no	yes	no	no	yes	yes	complex
4^∇^	71	male	no		2.5	45	37	no	yes	yes	no	yes	no	complex
5^∇^	65	male	no		2.0	43	33	no	yes	no	no	yes	yes	simple
6^∇^	53	male	yes		2.5	44	35	no	yes	yes	no	yes	yes	intermediate
7^∇^	77	male	yes		2.0	39	37	no	no	no	no	no	no	complex
8^∇^	73	male	no		2.0	39	34	no	no	no	no	no	no	complex
9^∇^	47	male	no		3.0	36	24	no	yes	no	yes	no	no	simple
10^∇^	58	male	no		3.0	42	34	no	yes	yes	no	no	no	intermediate
11	66	female	no		3.0	32	32	no	no	no	yes	yes	yes	simple
12	76	male	no		3.0	41	33	no	no	no	yes	yes	yes	intermediate
13	59	male	no		3.0	32	46	yes	yes	no	yes	yes	yes	complex
14	40	male	yes		2.0	32	32	no	yes	no	no	no	yes	intermediate
15	48	male	yes		2.0	35	33	no	yes	no	no	yes	yes	intermediate
16	71	female	no		4.0	33	34	no	yes	yes	yes	yes	yes	intermediate
17	66	male	yes		2.0	43	42	no	yes	yes	yes	no	yes	complex
18	58	male	yes		3.0	40	37	yes	yes	yes	no	yes	yes	complex
19	78	male	yes		2.0	39	38	yes	yes	no	yes	no	yes	intermediate
20	63	male	no		2.0	34	30	no	yes	no	no	no	no	simple
21	80	male	no		3.0	46	67	yes	no	no	yes	no	no	complex
22	76	female	no		3.0	37	34	no	yes	no	yes	yes	yes	simple
23	42	female	no		2.0	34	30	no	yes	yes	no	yes	no	intermediate
24	85	male	yes		3.0	37	36	no	no	no	no	no	yes	intermediate
25	55	male	no		30.	35	38	no	yes	no	yes	yes	no	intermediate
26^∇^	61	male	no		3.0	38	36	yes	yes	yes	yes	yes	no	complex
27^∇^	69	female	no		3.0	37	34	no	yes	no	yes	no	no	complex
28^∇^	68	female	yes		2.0	37	36	yes	yes	yes	no	yes	yes	intermediate
29^∇^	60	male	yes		3.0	35	35	no	yes	no	no	yes	yes	intermediate
30	47	male	yes		3.0	41	30	no	yes	no	yes	yes	yes	simple
31	66	female	no		2.0	42	37	no	yes	no	yes	Yes	yes	simple
32	62	male	no		2.0	38	35	no	yes	no	no	yes	yes	complex
33	36	female	no		2.0	31	30	no	yes	no	yes	yes	yes	simple
34	44	male	yes		2.5	36	35	no	yes	no	no	yes	no	complex
35	58	male	yes		3.0	40	34	no	yes	no	no	yes	yes	intermediate
36	63	male	no		3.0	30	32	no	yes	no	no	yes	no	intermediate
37	75	male	no		3.0	47	40	yes	yes	no	yes	yes	yes	intermediate
38	62	female	no		3.0	32	34	no	yes	no	no	yes	yes	simple
39^∇^	78	female	no		3.0	36	29	no	yes	no	no	yes	no	simple
40	79	female	no		2.0	33	25	no	yes	no	yes	yes	yes	simple

Abbreviations: 3D,three-dimensional, ∅_AscAo,_ diameter of ascending aorta; ∅_DescAo,_ diameter of descending aorta; AD, aortic dissection;

TL, true lumen; FL, false lumen; *defined according to ACC/AHA 2022 guidelines; ^∇^segmented by a medical student.

**Fig. 1:**
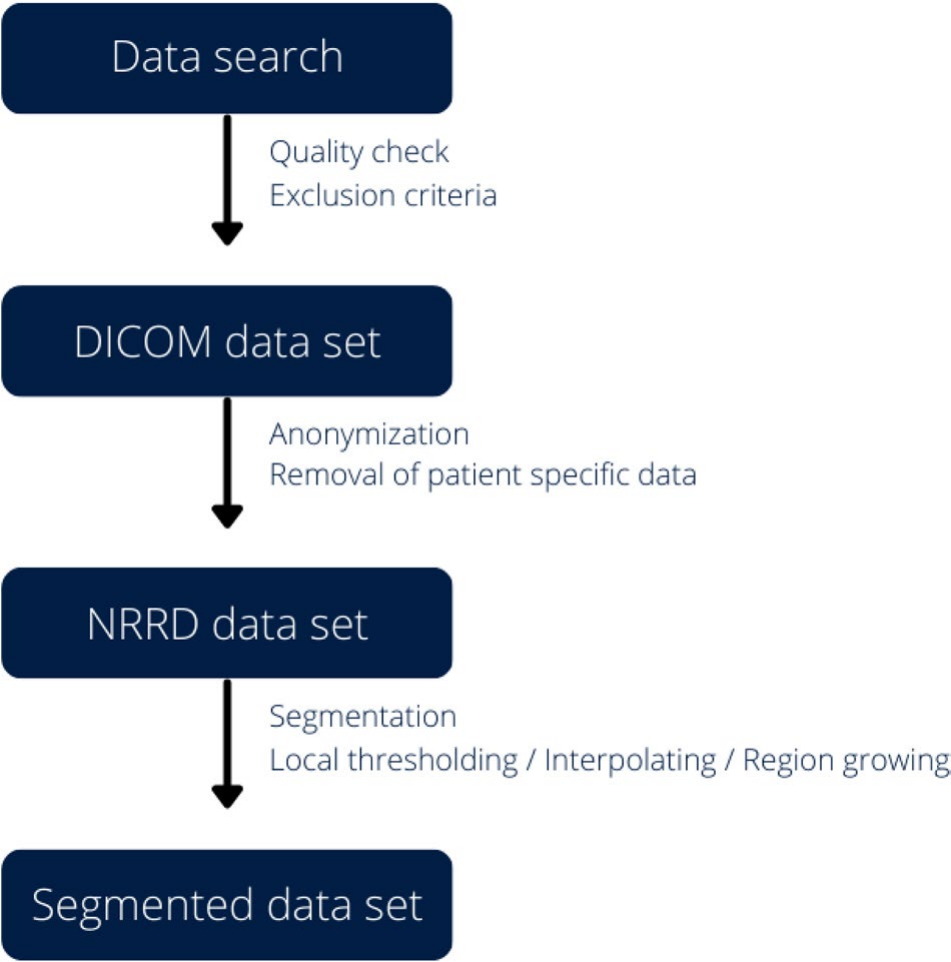
Workflow from initial data search to the creation of our final segmented data set.

After selecting the appropriate CTAs, all patient specific data was removed during conversion from Digital Imaging and Communications in Medicine (DICOM) format to Nearly Raw Raster Data (NRRD) format. Additionally for the purpose of complete anonymization, the head and facial regions were cropped from the CT-files, which, as already mentioned, waived the need for informed consent. The data acquisition and selection workflow is shown in Fig. 1.

### Ground truth segmentation

Segmentation has been performed by three cardiac surgery residents and one medical student (segmentations by the student are marked to demonstrate that there is no difference of software-users) in a semi-automatic fashion with 3D Slicer Version 5.0.3^28–31^. The segmentation workflow starts with selecting an aortic CTA (.nrrd file) and loading it into 3D Slicer. Then the curvature anisotropic diffusion plug-in is applied for removing noise but persevering the edges of the images^32^. The settings that were used in the plug-in were a conductance of 1.00, iterations of 7–10 and time step set to 0.0625. We then created separate segmentations for true and false lumina.

#### Method 1: Local thresholding

**Fig. 2:**
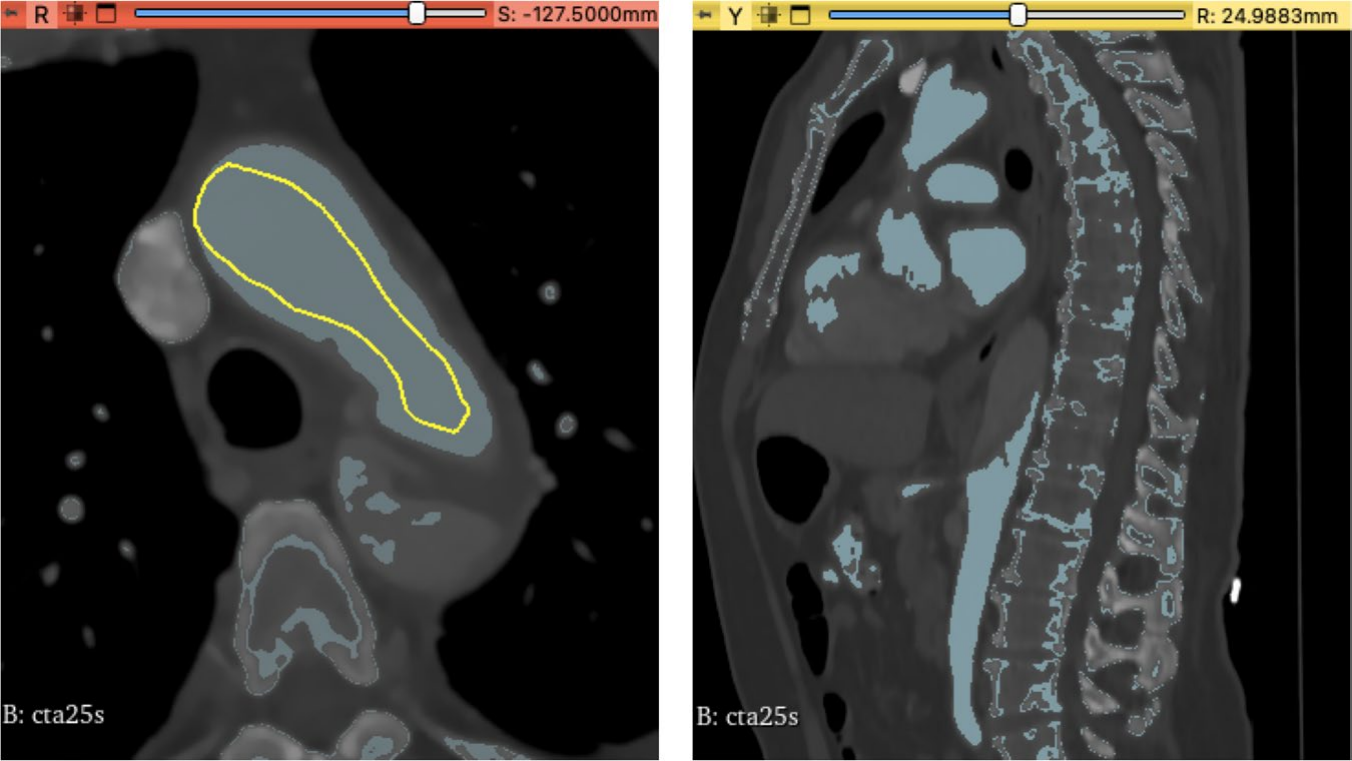
Local thresholding has been applied to the true lumen within the aortic arch of cta25s as seen here in the axial and sagittal plane. A light blue tone indicates regions to be part of the segmentation. As seen not all regions like bone structures or cardiac chambers have been mapped correctly therefore this method needed a lot of manual post-processing.

The first method we utilized was *local thresholding*, in which a local threshold range is manually specified through masking as seen in Figure 2. The segmentation algorithm we used for this was Grow Cut and the minimum diameter 3.0 mm. However due to threshold differences, noise, and artifacts, this method requires a lot of manual post-processing with paint and erase tools. This was described already for aortic aneurysms^33^, but as ADs are even more complex and artifact-rich anatomical structures this method reaches its limits in terms of usability. Nevertheless, it is still faster than performing a complete manual segmentation with paint and erase.

#### Method 2: Interpolating

An alternative approach was the *fill between slices* function. First, a manual segmentation is performed every five-to-eight slices. The function then interpolates the course of the target structure in between. This approach performed well on the long and straight parts of the vessels. In areas with multiple branching vessels, like in the aortic arch, the visceral- or iliac arteries, additional manual post-processing with paint and erase tools had to be done. Also, in the areas where the dissection flap originated or terminated, as well as in areas with higher variability of the flap, manual post-processing was needed. When the fill between slices is initialized in 3D Slicer, it first generates a preview of the segmentation. In this preview, one can already manually edit the slices in which the function does not provide a satisfactory result. The manually painted slices are then integrated in the function and it adapts live to it. This can be repeated until the whole segmentation tree is correctly segmented and by pressing ‘apply’ the function is finally executed. If there are still errors, manual post-processing with paint and erase can be done. The pre-selection before applying fill between slices can sometimes be eased by means of level tracing. This function tries to draw a closed path within the same intensity range back to the starting point and therefore recognizes the luminal borders automatically. This however, also works better in areas with only few artifacts. With this method, segmentation times were tendentially lower as reported in Table 2.

**Table 2:** Overview of the files and the used segmentation method.

Patient	Segmentation volume TL [cm3]	Segmentation volume FL [cm3]	Mean HU ± SD TL	Mean HU ± SD FL	Segmentation time [min]	Segmentation method
1^∇^	184,8	122,8	234 ± 34	322 ± 67	180	Local Thresholding
2^∇^	171,0	216,4	272 ± 39	278 ± 30	180	Local Thresholding
3^∇^	217,5	381,3	324 ± 58	221 ± 52	280	Local Thresholding
4^∇^	304,3	185,2	212 ± 30	108 ± 60	225	Interpolating
5^∇^	178,4	181,6	257 ± 45	256 ± 42	165	Interpolating
6^∇^	204,0	212,2	233 ± 60	231 ± 48	210	Local Thresholding
7^∇^	107,6	107,6	191 ± 45	191 ± 45	150	Local Thresholding
8^∇^	387,9	23,6	229 ± 32	78 ± 53	180	Interpolating
9^∇^	153,2	128,1	275 ± 27	251 ± 25	210	Local Thresholding
10^∇^	254,9	75,6	255 ± 44	178 ± 61	240	Local Thresholding
11	184,3	88,2	212 ± 35	202 ± 41	150	Region growing
12	315,0	124,7	366 ± 91	312 ± 60	145	Region growing
13	277,2	377,5	246 ± 42	241 ± 35	215	Region growing
14	136,5	130,4	331 ± 45	277 ± 70	135	Region growing
15	182,8	150,9	244 ± 34	164 ± 84	115	Region growing
16	181,8	110,4	352 ± 54	131 ± 89	55	Interpolating
17	254,1	358,2	281 ± 24	118 ± 80	80	Interpolating
18	331,5	279,2	229 ± 46	195 ± 44	70	Interpolating
19	381,31	24,2	283 ± 38	203 ± 31	70	Interpolating
20	191,6	80,3	388 ± 55	251 ± 111	50	Interpolating
21	363,6	439,0	271 ± 59	271 ± 35	85	Interpolating
22	278,8	164,9	235 ± 48	227 ± 46	40	Interpolating
23	202,6	125,4	402 ± 31	258 ± 77	290	Local thresholding
24	342,9	132,6	397 ± 54	284 ± 66	95	Interpolating
25	195,9	92,0	247 ± 22	202 ± 21	280	Local Thresholding
26^∇^	396,1	224,2	226 ± 33	267 ± 32	330	Local Thresholding
27^∇^	243,6	67,1	392 ± 52	392 ± 52	180	Local Thresholding
28^∇^	236,1	178,4	238 ± 32	107 ± 52	255	Local Thresholding
29^∇^	189,1	203,0	411 ± 53	315 ± 60	150	Local Thresholding
30	207,3	144,9	309 ± 39	291 ± 30	120	Interpolating
31	206,6	236,6	357 ± 56	280 ± 53	140	Interpolating
32	171,5	171,5	148 ± 26	148 ± 26	110	Interpolating
33	152,9	110,1	346 ± 35	260 ± 61	100	Interpolating
34	175,4	175,4	188 ± 25	188 ± 25	95	Interpolating
35	313,0	138,3	261 ± 54	121 ± 65	105	Region growing
36	212,0	111,0	160 ± 51	203 ± 50	130	Region growing
37	334,4	274,7	305 ± 40	241 ± 31	300	Interpolating
38	182,7	106,8	288 ± 50	260 ± 47	95	Region growing
39^∇^	216,0	104,9	513 ± 63	453 ± 65	150	Interpolating
40	214,1	54,6	424 ± 54	354 ± 38	105	Interpolating

It also shows segmentation volumes, voxels and segmentation times.

Abbreviations: TL, true lumen; FL, false lumen; HU, Hounsfield Units; SD, standard deviation.

^∇^segmented by a medical student.

#### Method 3: Region growing

The third alternative approach that we used utilized the *grow from seeds* function. With the paint function manual seeds are placed in each region that should belong to a separate segment. In this case true lumen, false lumen and others. Then the function is initialized, and a preview is shown. Areas that are not correctly segmented in the preview can be corrected by switching to manual paint mode and placing additional seeds in the misclassified region. The full segmentation is updated, and when satisfactory, the function can be finally executed by pressing ‘apply’. Manual post-processing with paint and erase is still required here most of the time to attain the desired result.

#### Aortic branches

**Fig. 3:**
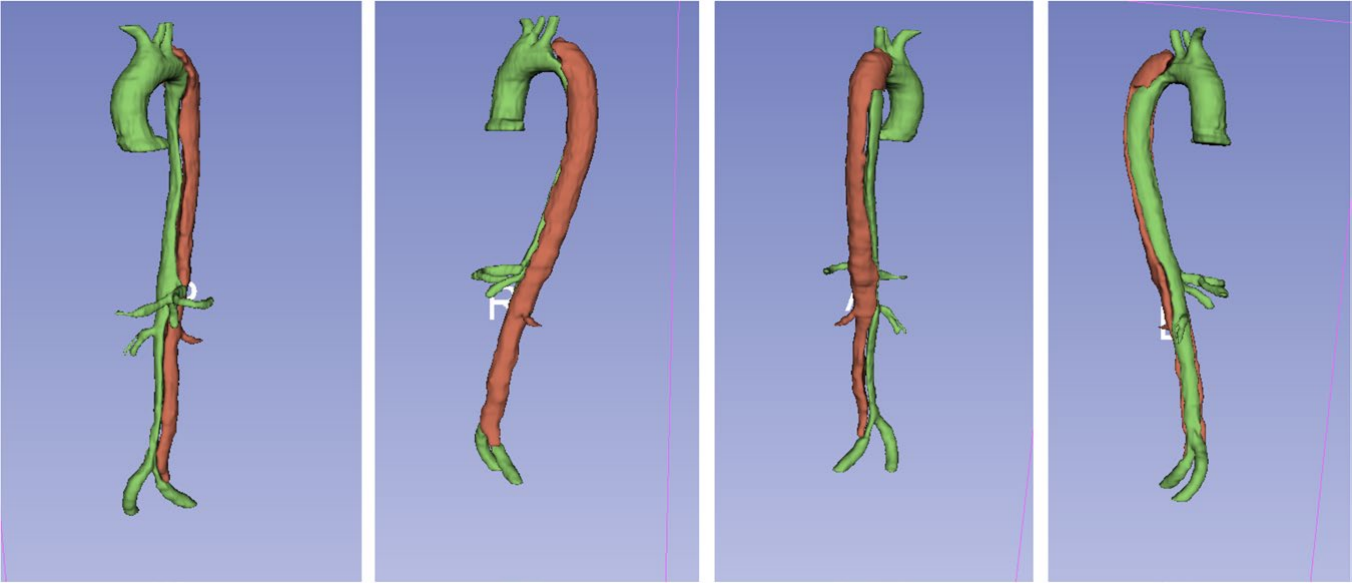
Segmented aortic dissection cta33s in 3D from different angles.

The most prominent supraaortal, visceral, and iliac arteries were also segmented mainly for orientation. The vessels that have been segmented were - from proximal to distal: the brachiocephalic trunc, the left carotid artery, the left subclavian artery, the celiac trunk, the superior mesenteric artery, the left and right renal arteries and the iliac arteries as seen in Figure 3. Depending on the segmentation method, a variable length of them is included in our segmentations. Most of them had to be manuall (re-)drawn in the segmentation process. Having perfectly segmented branching vessels was not the main focus of this study, that primarily was on exact aortic and AD morphology

#### Thrombus, Calcifications and artifacts

Clearly thrombosed sections have not been segmented. Darker areas with fluctuating contrast, where distinguishing a thrombus was not so clear, were attributed to late-filling artifacts and therefore segmented as lumen.

Calcifications and atherosclerotic areas were not included and left unsegmented.

Artifacts were segmented as lumen following the natural border of the vessel, since segmentations should represent the real dimensions of the vessel.

#### Segmentation times

In general segmentations performed with interpolating or region growing had shorter segmentation times. The segmentation times for local thresholding were an average time of 226 minutes, with a minimum of 150 minutes and a maximum of 330 minutes. For interpolating, the respective times were 118, 40 and 300 and for region growing 136, 95 and 215 minutes. Segmentation times, methods and volumes can be seen in Table 2.

#### Manual post-processing: Optimization for AI-applications

All aforementioned segmentation methods required manual post-processing with paint and erase on a slice by slice basis. Even though the semi-automated segmentations were suitable for clinical visualization of the dissection morphology, they were not precise enough for AI applications. Manual post processing required between two to three hours per segmentation. This time is not included in Table 2 with the segmentation times.

## Data Records

**Fig. 4:**
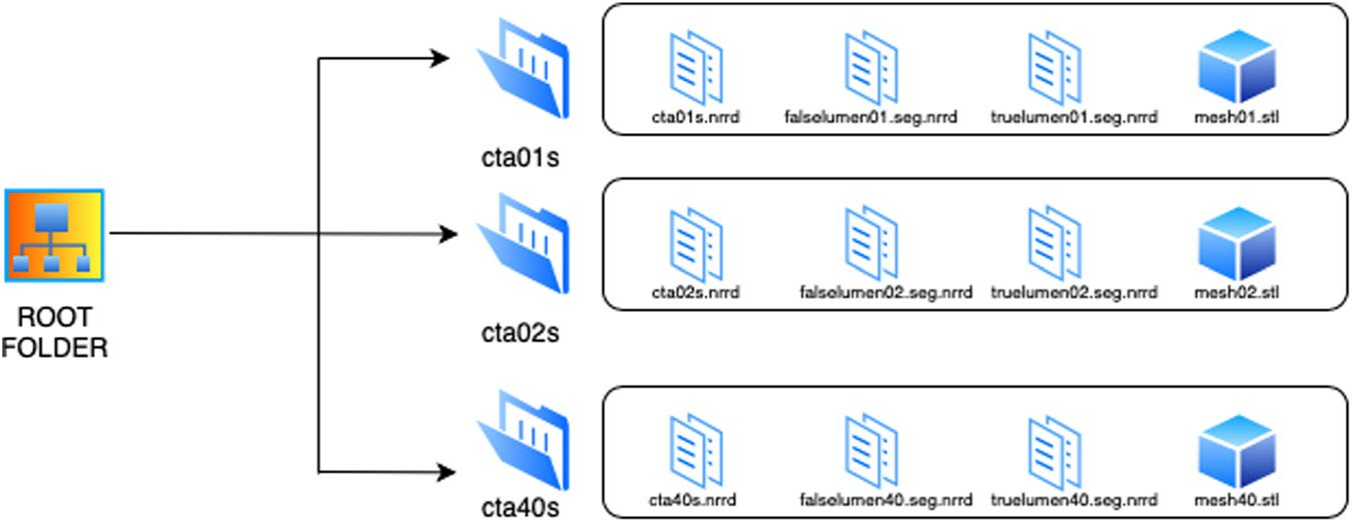
Folder structure of our dataset.

The 40 segmented CT scans are stored in a private figshare repository^34^ and can be downloaded freely. The anonymized CT scans are stored as single NNRD files. The segmentations are also provided as NRRD files depicting the true lumen and the false lumen. A surface mesh representation of the overall pathology is provided as Standard Triangle Language (STL) files. For convenience the data sets are stored in separate folders with their corresponding patient ID numbers (cta1, cta2, cta3, etc.) containing the files. Figure 4 shows the folder structure of our data set. Additionally, for giving a preview, there are video files (.mp4) of every segmentation in 3D with corresponding ID numbers (1, 2, 3, etc.) stored in a separate folder. The original preprocessed CT scans are also stored in a separate folder with their corresponding ID numbers (cta1, cta2, cta3, etc.).

The CT scans and segmentations can be cross-referenced against data Tables 1, 2 using their corresponding patient identification number. The Tables 1, 2 contain basic anatomical information like diameters and AD shape description, technical parameters, imaging data like slice thickness, segmentation volume and processing data such as segmentation times and methods.

We only used high quality CTAs with a resolution of 512 × 512 pixels and an average slice thickness of 2,56 mm. Only one scan had a slice thickness of 4 mm (cta16), all others were 3 mm or below. Most scans (n = 35) were reconstructed with overlapping slices (slice increment smaller than slice thickness). The routine CTA data are not isotropic (meaning voxels do not have equal side lengths, e.g. 1x1x1 mm), which might be a limitation for 3D analysis. All CTAs have been done in the arterial contrast phase to guarantee optimal visualization of the dissected aorta. However, due to delayed filing of the false lumen, differentiation of a thrombus can be challenging sometimes. Locating entries and reentries was not performed for this study. The CTAs were not ECG-gated, as this was not routinely done at our institution for this indication.

## Technical Validation

All of the used CT data was acquired during routine clinical practice with medical CT scanners, which as qualified medical products routinely undergo maintenance and quality control under the responsibility of a qualified medical physicist^35^. In Table 1 an overview over the slice thickness of the used CTs data is shown.

## Usage Notes

Our shared collection can be freely downloaded. Furthermore, the data provided is free to share, modify, transform, remix or copy and redistribute in any medium or format. The data within this work is licensed under a Creative Commons Attribution 4.0 International License.

The NRRD files can be accessed and processed through open-source medical imaging platforms like 3D Slicer^28,31^ and MeVisLab^36^. Additionally, a variety of advanced commercial software products are available. As STL files are widely used for 3D printing, the provided imaging data can be directly used for printing real life models of aortic dissections too^37^ and are often a preliminary step for fluid dynamics simulations^38^.

Finally, we want to encourage researchers to use this unique data set for their work to develop tools for aiding clinicians in their everyday practice diagnosing and treating patients and understanding aortic dissections in a more comprehensive way. To our knowledge this is the first publicly available dataset for aortic segementations of this scope that has been created, validated and provided by clinical experts in the field.

## Data Availability

All the data has been generated using the functionalities of 3D Slicer (https://www.slicer.org/). The surface meshes (.stl) have been generated using a Python implementation based on TorchIO (https://torchio.readthedocs.io/) and PyACVD (https://github.com/pyvista/pyacvd). The implementation is freely available at https://github.com/apepe91/AD_NRRD_TO_STL.
